# Neuroinflammation as measured by positron emission tomography in patients with recent onset and established schizophrenia: implications for immune pathogenesis

**DOI:** 10.1038/s41380-020-0829-y

**Published:** 2020-06-30

**Authors:** Silke Conen, Catherine J. Gregory, Rainer Hinz, Richard Smallman, Fabiana Corsi-Zuelli, Bill Deakin, Peter S. Talbot

**Affiliations:** 1 Division of Neuroscience and Experimental Psychology, University of Manchester, Manchester Academic Health Science Centre, Manchester, M13 9PT UK; 2 Division of Informatics, Imaging & Data Sciences, University of Manchester, University of Manchester, Manchester Academic Health Science Centre, Manchester, M13 9PL UK; 3 Division of Psychiatry, University of São Paulo (FMRP - USP), São Paulo, 14048-900 Brazil

**Keywords:** Neuroscience, Molecular biology, Schizophrenia

## Abstract

Positron emission tomography (PET) imaging of the 18 kDa translocator protein (TSPO), which is upregulated in activated microglia, is a method for investigating whether immune activation is evident in the brain of adults with schizophrenia. This study aimed to measure TSPO availability in the largest patient group to date, and to compare it between patients with recent onset (ROS) and established (ES) schizophrenia. In total, 20 ROS patients (14 male), 21 ES (13 male), and 21 healthy controls completed the study. Patients were predominantly antipsychotic-medicated. Participants underwent a PET scan using the TSPO-specific radioligand [^11^C](R)-PK11195. The primary outcome was binding potential (BP_ND_) in the anterior cingulate cortex (ACC). Secondary outcomes were BP_ND_ in six other regions. Correlations were investigated between TSPO availability and symptom severity. Data showed that mean BP_ND_ was higher in older (ES and controls) compared with younger (ROS and controls) individuals, but did not significantly differ between ROS or ES and their respective age-matched controls (ACC; ANOVA main effect of diagnosis: *F*_1,58_ = 0.407, *p* = 0.526). Compared with controls, BP_ND_ was lower in antipsychotic-free (*n* = 6), but not in medicated, ROS patients. BP_ND_ in the ES group was negatively correlated with positive symptoms, and positively correlated with negative symptom score. Our data suggest ageing is associated with higher TSPO but a diagnosis of schizophrenia is not. Rather, subnormal TSPO levels in drug-free recent-onset patients may imply impaired microglial development and/or function, which is counteracted by antipsychotic treatment. The development of novel radioligands for specific immune-mechanisms is needed for further clarification.

## Introduction

Several lines of evidence suggest that immune activation and inflammation play a role in the pathophysiology of schizophrenia. For example, meta-analyses report increased blood levels of c-reactive protein, IL-6 and a range of other cytokines compared with non-patients and some changes normalise with recovery [1, 2]. However, increases are rarely in the pathological range, and conventional anti-inflammatory drugs such as aspirin have limited efficacy.

In the last decade, it has become possible to investigate inflammation in the living human using positron emission tomography (PET) in various neurodegenerative disorders. Microglia are the resident macrophages of the central nervous system (CNS) [3]. In their activated immune state, microglia secrete cytokines and chemokines and this is associated with increased expression of the translocator protein (TSPO) for which a number of radioligands have been developed.

Early studies reported increased brain TSPO expression in schizophrenia, suggesting the presence of microglial activation and neuroinflammation [4, 5]. These studies motivated our initial PET study using [^11^C]*(R)-*PK11195, which found no difference in TSPO expression between medication-free schizophrenia patients and matched healthy subjects [6]. Most other studies have reported unchanged or decreased TSPO expression [6–13]. Indeed, a recent meta-analysis of five studies concluded that PET TSPO radioligand binding is statistically significantly reduced in recent onset schizophrenia unrelated to treatment status [14].

In the present study, we aimed to compare TSPO expression between (i) schizophrenia patients with recent onset of symptoms (ROS group); (ii) an established illness group with at least 10 years of symptoms (ES group); and (iii) healthy controls using [^11^C]*(R)-*PK11195 PET in order to determine differences across illness stages and to get a more conclusive answer regarding a potential role of microglial activation in schizophrenia. The primary region of interest (ROI) was the anterior cingulate cortex (ACC), based on evidence of grey matter loss [15] and increased glutamate content in ACC [16]. Secondary ROIs were based on our previous research [6] and included the prefrontal cortex (PFC), orbitofrontal cortex (OFC), parietal cortex, temporal cortex, caudate, putamen, thalamus, amygdala, hippocampus, and brainstem.

### Subjects and methods

#### Subjects

In total, 20 recent onset patients (14 males; mean ± standard deviation [SD]: 24.2 ± 5.1 years); 21 established patients (13 males; mean ± SD: 46.0 ± 6.0 years); and 21 age- and sex-matched controls: 10 matched to recent onset (8 males; mean ± SD: 25.5 ± 4.1 years), 11 matched to established patients (7 males; mean ± SD: 47.0 ± 5.0 years) completed the study. It was calculated that a study sample size of 20 participants in each group could detect a between-group difference in binding potential (BP_ND_) of 15%, with a power of 80%, *α* = 0.05, two-sided. Patients met DSM-IV criteria for schizophrenia, schizoaffective disorder or schizophreniform disorder (all referred to in the paper as “schizophrenia”), as determined by consensus of two clinicians (CG, PST, and/or BD) following case note review and patient interview.

Inclusion criteria for recent onset patients were: <5 years from onset and <12 weeks lifetime exposure to antipsychotic medication, and for established patients >10 years from onset and no major changes to antipsychotic medication over the previous 8 weeks. Controls were age- and sex-matched to patients and included if they had no personal or first-degree relative with a history of psychosis, or a history of depression within the past two years. We excluded participants who: had significant physical illness such as an inflammatory or neurological condition; were taking anti-inflammatory medication; and who met DSM-IV criteria for harmful use of substances within the past month or dependence within the past 6 months. Females could not participate when pregnant or breast-feeding.

For patients, symptom severity was assessed on the day of scanning using the Positive and Negative Syndrome Scale (PANSS) [17] and level of functioning by the Personal and Social Performance Scale (PSP) [18]. Scales were administered and scored by a trained clinician (CG).

The present study was conducted at the University of Manchester (UoM), as part of a multi-centre multi-modal imaging project (the SPRING study) that was conducted at UoM, the University of Nottingham and Cardiff University. The study was approved by the North-West Lancaster National Research Ethics Service Committee and the United Kingdom Administration of Radioactive Substances Advisory Committee, and was carried out in compliance with the Declaration of Helsinki (1964), amended in Edinburgh (2000). Participants gave their written informed consent prior to study participation.

#### Image acquisition

Image acquisition and analysis methodology for this study was similar to recent publications from our group [6, 19]. In summary, a high-resolution T_1_-weighted magnetic resonance (MR) image was acquired for data-processing, identification of ROIs, and voxel-based morphometry analysis using a 3T Philips Achieva scanner. PET imaging was performed using a High-Resolution Research Tomograph (Siemens). For each PET scan, following a 6 minute transmission scan, [^11^C]*(R)-*PK11195 was intravenously injected with a target dose of 740MBq. Dynamic emission data were acquired for 60 min. The data was re-binned into 18 frames of variable duration and reconstructed.

#### Image processing

A summation image was created and co-registered with the T_1_-weighted MR image using Vinci version 2.55 (Max-Planck Institute for Neurological Research, Cologne, Germany), segmented into grey and white matter using SPM8 (Statistical Parametric Mapping, Wellcome Department of Imaging Neuroscience, Institute of Neurology, London, UK), and normalised using the n30r83 version of the probabilistic brain-atlas by Hammers et al. [20].

A binary grey matter mask was created in Vinci using a threshold of 0.5. Object maps (standardised anatomical areas) were defined using Analyze 11.0 (BIR, Mayo Clinic, Rochester, MN). Parametric images of BP_ND_ were created using MICKpm 5.2 (in-house MATLAB based software), using the simplified reference tissue model and the time activity curve from the bilateral grey matter cerebellum as reference tissue input function. BP_ND_ for each ROI were read out from the parametric maps by applying the object maps.

#### Statistical analysis

We used SPSS Statistics Version 22 (IBM, Armonk, NY, USA). Data were analysed by SC, CG, and RH. Differences in demographic and clinical characteristics between groups were assessed using independent samples *t*-tests (2-sided), chi-square test of homogeneity or Fisher’s exact test (two-sided). Between-group differences in BP_ND_ in the ACC were assessed using a two-way ANOVA with diagnosis (patients and controls) and age (younger and older) as fixed factors. Between-group differences in BP_ND_ in all ROIs (the secondary ROIs) were assessed using repeated-measures ANOVA with brain region (ACC, PFC, OFC, parietal cortex, putamen, thalamus, and brainstem) as the within-subjects factor. The effect size of between-group differences in BP_ND_ was calculated as Cohen’s *d* (the difference between the means divided by the pooled standard deviation).

Shapiro-Wilk’s test and visual inspection of Q-Q plots was done to assess normal distribution and possible outliers. Box’s test was done to assess equality of covariance, Levine’s test to assess homogeneity of variances, and Mauchly’s test to assess sphericity of the data using the Greenhouse-Geisser correction if violated. Differences in individual ROIs were assessed post hoc in an exploratory fashion using independent samples *t*-tests.

Correlation of regional BP_ND_ with PANSS and PSP scores were assessed using Pearson’s r, or Spearman’s coefficients if a measure was not normally distributed. Findings were considered significant at the *p* < 0.05 (2-sided) level.

## Results

Demographic and clinical characteristics for all participants are outlined in Table 1. An overview of psychotropic medication of the patient groups can be found in the supplement, Tables S1 and S2. Patients and controls were well matched with no significant differences between them in age, sex, BMI or injected mass of [^11^C]*(R)-*PK11195. A greater proportion of patients were smokers compared with controls. There were no significant differences between recent onset and established patients in PANSS scores, PSP scores, sex or smoking status (Table 1). Established patients had a significantly higher BMI than recent onset patients and, by design, established patients were older and had a significantly longer duration of illness. There was no difference between the patient groups in antidepressant usage. Combining all patients and all controls there was no main effect of sex, BMI, smoking status or injected mass of [^11^C]*(R)-*PK11195 so these were not included as covariates.

**Table 1:** Demographic and clinical characteristics of participants.

	Recent			Established			Recent vs ES
Characteristics	Controls (*n* = 10)	Patients (*n* = 20)	*p*	Controls (*n* = 11)	Patients (*n* = 21)	*p*	*p*
Sex (M)	8 (80%)	14 (70%)	–	7 (64%)	13 (62%)	–	–
Age (years)	25.5 ± 4.1	24.2 ± 5.1	0.48	46.7 ± 4.8	46.3 ± 6.4	0.85	0.000*
BMI (kg/m^2^)	26.5 ± 3.6	24.7 ± 3.6	0.19	26.6 ± 5.2	29.5 ± 5.3	0.15	0.002*
Smoking (Yes)	4 (40%)	17 (85%)	–	2 (18%)	15 (71%)	–	–
Duration of illness (months)	–	16 ± 17	–	–	249 ± 88	–	0.000*
PANSS total	–	57 ± 12	–	–	55 ± 13	–	0.74
PANSS positive	–	15 ± 6	–	–	15 ± 7	–	0.92
PANSS negative	–	12 ± 4	–	–	13 ± 5	–	0.53
PANSS general	–	30 ± 6	–	–	27 ± 6	–	0.22
PSP	–	59 ± 14	–	–	53 ± 15	–	0.14
Injected mass of PK11195 (μg)	1.39 ± 0.63	1.86 ± 1.72	0.29	1.28 ± 0.66	1.22 ± 0.91	0.84	0.15

Values are presented as mean ± SD.

*BMI* body mass index, *PANSS* positive and negative syndrome scale, *PSP p*ersonal and social performance scale.

*Significant difference between groups at *p* ≤ 0.05.

Mean BP_ND_ for caudate, amygdala, hippocampus and temporal cortex was around zero (see Supplementary Fig. S1) and these regions were therefore not included in subsequent analyses. The primary dependent variable was BP_ND_ in the ACC.

Furthermore, in the supplementary material, data is presented of exploratory *post hoc* analyses that were carried out to test the significance of any difference in BP_ND_ in individual ROIs between our patient groups and matched controls, and to establish any main effect of age or age X diagnosis interaction (Supplementary Table S3, Fig. S2, and Table S4).

**Fig. 1:**
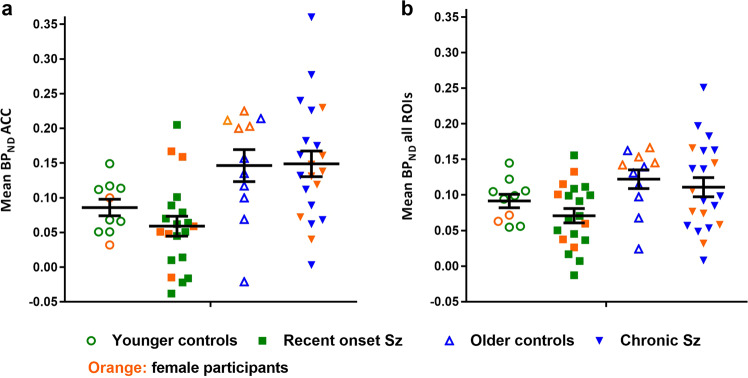
Mean [^11^C]*(R)-*PK11195 BP_ND_ in recent onset and established (chronic) schizophrenia (Sz) patients. Patient binding potential is compared with age and sex-matched controls in the anterior cingulate cortex (ACC; [**a**]) and in all regions of interest (ROIs) together (i.e., ACC, PFC, OFC, parietal cortex, putamen, thalamus and brainstem; [**b**]). Orange symbols represent female participants. Black lines in scatterplot indicate mean ± SEM per group.

Visual inspection of the data showed that BP_ND_ in the ACC was similar between each patient group and its respective control group (Fig. 1a). BP_ND_ was greater in established patients and their controls compared with the younger group of recent onset patients and controls. Two-way ANOVA showed no significant difference in BP_ND_ between patients and controls (no main effect of diagnosis; *F*_1,58_ = 0.41, *p* = 0.53). There was a significant main effect of age (*F*_1,58_ = 15.49, *p* < 0.001) but no significant age*diagnosis interaction (*F*_1,58_ = 0.59, *p* = 0.45).

Statistical effects of age and diagnosis were similar to ACC across all 7 ROIs (Fig. 1b) with no main effect of diagnosis (*F*_1,58_ = 1.45, *p* = 0.23), a main effect of age (*F*_1,58_ = 7.05, *p* = 0.01), and no significant age*diagnosis interaction (*F*_1,58_ = 0.13, *p* = 0.72). However, there was a significant ROI*diagnosis interaction (*F*_3.152,182.787_ = 2.97, *p* = 0.03) due to statistically significant overall reductions in putamen and thalamus in patients compared with controls (Supplementary Fig. S3 and Table S5). The ROI*diagnosis*age interaction was not significant (*F*_3.152,182.78_ = 1.90, *p* = 0.13).

**Fig. 2:**
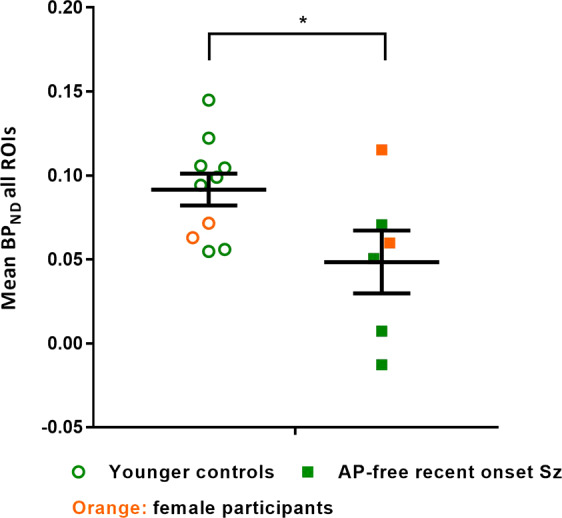
Mean [^11^C]*(R)-*PK11195 BP_ND_ in antipsychotic-free recent onset schizophrenia (Sz) patients. Patient binding potential is compared with age and sex-matched controls in all regions of interests combined (ROIs). Orange symbols represent female participants. Black lines in scatterplot indicate mean ± SEM per group.

Patients with recent onset schizophrenia who were antipsychotic-free (*n* = 6) were compared with those who were currently taking an antipsychotic (*n* = 14; an overview of their patient characteristics and the matched controls characteristics can be found in Supplementary Table S6). Mean BP_ND_ across all ROIs combined was lower in antipsychotic-free patients compared with controls (Fig. 2, Table 2) producing a statistically significant main effect of group (*F*_1,14_ = 5.26, *p* = 0.04). There was no region*group interaction (*F*_(2.043,28.604)_ = 0.27, *p* = 0.77). The data for individual ROIs (Table 2) showed that BP_ND_ was lower in antipsychotic-free patients compared with controls, with medium (≥0.5) or large (≥0.8) effect sizes in all ROIs. There were no statistical differences in BP_ND_ between antipsychotic-exposed patients and their recent onset controls (Supplementary Table S7), or between antipsychotic-free and exposed recent-onset patients (Supplementary Fig. S4 and Table S8). Except in the brainstem, where BP_ND_ was higher in antipsychotic-exposed compared with antipsychotic-free patients.

**Table 2:** Regional [^11^C]*(R)*-PK11195 BP_ND_ in antipsychotic-free recent onset schizophrenia patients compared with age- and sex-matched healthy controls.

	Controls (*n* = 10)	Antipsychotic-free patients (*n* = 6)	Controls vs Antipsychotic-free	
Region	BP_ND_	BP_ND_	Difference (%)	Cohen’s *d*
ACC	0.086 ± 0.038	0.036 ± 0.073	−58	0.86
PFC	0.058 ± 0.042	0.024 ± 0.071	−59	0.58
OFC	0.039 ± 0.035	0.0003 ± 0.064	−99	0.75
Parietal	0.044 ± 0.041	0.020 ± 0.052	−55	0.51
Putamen	0.075 ± 0.047	0.018 ± 0.042	−76	1.28
Thalamus	0.196 ± 0.063	0.144 ± 0.077	−27	0.74
Brainstem	0.142 ± 0.061	0.097 ± 0.058	−32	0.76
Mean all ROIs	0.092 ± 0.030	0.048 ± 0.046	−48	1.13

BP_ND_ values are presented as mean ± SD.

*BP*_*ND*_ binding potential, *ROI* region of interest, *ACC* anterior cingulate cortex, *PFC* prefrontal cortex, *OFC* orbitofrontal cortex.

**Fig. 3:**
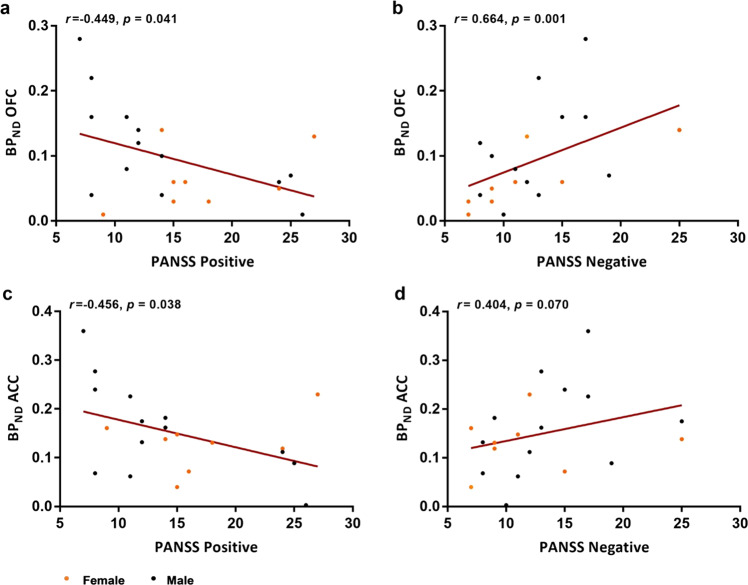
Correlation of Positive and Negative Syndrome Scale (PANSS) scores and binding potential in established schizophrenia patients. Correlation between [^11^C]*(R)-*PK11195 binding potential (BP_ND_) and scores on the positive (**a**) and negative (**b**) subscales of the  PANSS in the orbitofrontal cortex (OFC), and scores on the positive (**c**) and negative (**d**) subscales of the PANSS in the anterior cingulate cortex (ACC) across established schizophrenia patients, divided into male and female participants.

In the established patients, there was a moderate negative correlation between PANSS positive score and [^11^C]*(R)*-PK11195 BP_ND_ in all ROIs except in the brainstem, reaching significance in the ACC, PFC, OFC and putamen (Supplementary Table S9). In contrast, PANSS negative scores positively correlated with BP_ND_ in all ROIs, reaching significance in the PFC and OFC. Figure 3 presents the correlation of PANSS positive and negative scores in the OCC, which showed the strongest correlation, and in the ACC, the primary ROI. PANSS general and total scores and the PSP did not correlate with BP_ND_ in any region (Supplementary Table S9). The correlation between BP_ND_ and clinical measures in patients with recent onset schizophrenia and all patients with schizophrenia can be found in the supplement, Tables S10 and S11, respectively.

A voxel-based morphometry analysis using SPM12 was carried out on the T_1_-weighted MR images to examine potential differences in grey matter volume between schizophrenia patients and controls. No significant between-group grey matter volume differences were found, suggesting that the significant differences in BP_ND_ in our data are unlikely to be an artefact of differences in regional tissue volumes. For details of the VBM methodology, please see Holmes et al. [19].

## Discussion

The present study was conducted to determine whether there are differences in regional brain TSPO expression, an indication of microglial activation, across schizophrenia illness stages, and is the largest PET study to investigate potential microglial activation in schizophrenia to date. In the ACC, the primary ROI, there was no main effect of diagnosis and neither the recent onset nor the established group differed from their respective controls in [^11^C](*R*)-PK11195 BP_ND_. Similarly there was no main effect of group across all 7 ROIs (i.e., ACC, PFC, PFC, parietal cortex, putamen, thalamus and brainstem) but 2 ROIs differed significantly from the others; in the thalamus and putamen, [^11^C](*R*)-PK11195 binding was reduced in the total sample of 40 patients compared with the 20 controls. There was an increase in the older clinical and control groups compared with the younger groups, in line with a previous study using TSPO radioligand [^11^C]PBR28 [21]. The recent onset group tended to have reduced [^11^C](*R*)-PK11195 binding compared with their controls and the reduction was statistically significant in the 6 patients who were not receiving antipsychotics. This pattern of results was repeated in the 6 other secondary cortical and subcortical ROIs in the recent onset patients.

Our study has several limitations. [^11^C]*(R)*-PK11195 has a relatively poor signal to noise ratio [22] which limits the ability to detect small changes in TSPO binding as might occur in a low level inflammatory condition like schizophrenia. Nevertheless, this does not account for the significant reduction in binding seen in antipsychotic-free patients. The choice of radioligand and analytic methods are discussed in the supplementary material. Having a patient population comprising of schizophrenia, schizoaffective and schizophreniform disorder may contribute to heterogeneity. Currently, there is however no detailed knowledge about neurological differences between these patient groups and we therefore decided not to differentiate between them. Furthermore, clinically significant depressive symptoms might have introduced a tendency towards elevated [^11^C](*R*)-PK11195 BP_ND_ as shown in previous studies in major depressive disorder [19], yet we did not assess depressive symptoms by means of depression scales. Our results however indicate an absence of increased PET TSPO binding in patients, suggesting no increase was caused by potential depression comorbidity. Our study included relatively few antipsychotic-free patients (*n* = 6), of whom only two were antipsychotic-naïve. Although all our patients with recent onset schizophrenia had <12 weeks exposure to antipsychotics, our results suggest that even minimal exposure to antipsychotics may have an influence on BP_ND_. Established patients had a long history of exposure to antipsychotic medication, making it difficult to draw firm conclusions about the effects of illness duration. Our patients were also mainly stable with mild symptom severity, limiting the ability to detect correlations between severity and BP_ND_. It is possible that increased TSPO expression can be found in the prodrome or at the onset of symptoms or in those with treatment resistant schizophrenia. Finally, our study was not designed or powered to investigate between-sex differences in BP_ND_. Visual inspection of Fig. 2, in which antipsychotic-free recent onset patients show significantly lower BP_ND_ than matched younger controls, suggests that this difference is driven by the male patients. However, the very small number of females in each group (*n* = 2) makes it impossible to draw clear conclusions and future studies could investigate this possibility further.

We did not find evidence of increased microglial activation in recent onset patients with minimal (<12 weeks) or no exposure to antipsychotic drugs. The results are in keeping with the majority of previous studies [8–13] also showing no TSPO difference between patients and controls, and a recent post-mortem study showing no increase in TSPO in brain tissue of schizophrenia patients [23]. One major study reported increased [^11^C]PBR28 TSPO binding in people with ultra-high risk for psychosis and with early psychosis [24]. However, the analysis method has been challenged [25]. The data were included in a recent meta-analysis that found no increase in TSPO binding in schizophrenia regardless of disease stage, but on the contrary found strong evidence for reductions that were unrelated to drug treatment [14]. In the present study, we found that BP_ND_ was lower in antipsychotic-free recent onset patients across all ROIs compared with matched controls as have other studies [7, 9, 10]. This may potentially reflect a chronic neurodevelopmental abnormality of microglial function in schizophrenia. It is therefore possible that TSPO availability does not accurately reflect low level inflammation in schizophrenia, or that it reflects a reduced ability by the immune system to mount an anti-inflammatory response. Recent studies report promising PET tracers for the quantification of mitochondrial and synaptic function, which in the future might be preferable over TSPO tracers [26].

We also found reduction in all patients compared with controls in the putamen and thalamus in the entire sample. Our findings therefore add to an emerging body of evidence that TSPO availability is reduced in the early years of psychosis and possibly especially apparent in drug-free patients. The fact that reduced BP_ND_ was also associated with greater PANSS positive symptom scores in those with many years of illness suggests reduced BP_ND_ could be a marker close to an underlying pathogenic mechanism of psychosis.

Reduced TSPO binding in schizophrenia stands in contrast to increases in PET TSPO binding reported in several CNS inflammatory disorders with known pathologies involving microglial activation including stroke, multiple sclerosis, and Alzheimer’s and Huntington’s diseases. Furthermore, we and one other group have recently reported increases in [^11^C](*R*)-PK11195 binding in patients with major depression which is associated with mild increases in systemic immune signalling although not with gross CNS inflammatory pathology [19, 27]. Nevertheless, the anti-microglial anti-inflammatory antibiotic, minocycline showed evidence of efficacy in depression in two clinical trials (see [28]), suggesting the raised PET TSPO binding could reflect a causal inflammatory process and possibly microgliosis. In contrast, minocycline was of no benefit in a recent major clinical trial in recent onset schizophrenia, in keeping with the absence of increased PET TSPO binding [28].

A neuroinflammatory process is however not ruled out by the present results. For example, in an infection-mediated neurodevelopmental mouse model of schizophrenia, behavioural abnormalities were associated with raised cytokine concentrations in the PFC but with reduced prefrontal TSPO concentrations with no microglial activation [29]. Furthermore, much of the evidence that inflamed microglia produce increased TSPO expression comes from in vitro rodent studies, but there is evidence that human and rodent microglia may respond differently to immune activation in vitro [30]. Indeed, three recent expression-profiling studies in several post-mortem brain datasets, report no evidence of increased microglial patterns of gene-expression in schizophrenia [31–33]. The same studies do, however, report increased astroglial gene expression which could mediate neuronal dysfunction in schizophrenia. Importantly for the present discussion, it is known that astroglia are a principle source of transforming growth factor β in the brain, which has potent effects in shifting microglia into their non-amoeboid phenotype associated with their important role in synaptic remodelling [34–36]. Reduced TSPO binding in schizophrenia could thus reflect an increase in the proportion of microglia that are in a low TSPO-expressing synaptic housekeeping state; this shift could be a cause or an effect of the recent evidence for an old hypothesis that excessive synaptic elimination occurs in schizophrenia [37, 38].

Recent onset patients on medication showed no reductions in BP_ND_ and this could reflect a normalisation of the process causing the reduction in the drug-free patients or a pro-inflammatory effect of the drugs. There is little evidence of pro-inflammatory effects of antipsychotic drugs in terms of cytokine levels in brains from treated animals or in clinical populations. In the current study, neither lifetime exposure to medication nor current exposure correlated with BP_ND_ or symptom ratings (data not shown) but the selection criteria resulted in little variation in treatment exposure within the two clinical groups. In the established group greater BP_ND_ correlated with greater negative symptom scores as in our previous study [6]. But lower BP_ND_ values were associated with positive symptoms. Positive and negative symptom ratings were, as expected, orthogonal (*r* = 0.02, *n* = 41). The findings imply that the two syndromes have an independent pathogenesis which bear an opposite relationship with TSPO expression. The outcome of these two independent processes (the ratio of positive to negative symptoms) determined a remarkable 27–40% of the variance in observed BP_ND_ in the three cortical regions.

In conclusion, we report that schizophrenia is not associated with microglial activation as measured by TSPO expression in the early or established phases of the illness. Indeed, reduced TSPO expression was seen in the putamen and thalamus, in antipsychotic-free recent onset patients and in association with positive symptoms in established illness. The results add to a growing body of evidence that reduced TSPO availability may reflect an important aspect of pathogenesis. However, understanding this signal requires the development of PET tracers for the different molecular and cellular components of neuroinflammation that converge to produce the pattern of TSPO expression reported in schizophrenia.

## Supplementary information

Supplement
