# Supplementary material for: Neuroinflammation as measured by positron emission tomography in patients with recent onset and established schizophrenia: implications for immune pathogenesis
Source: Mol Psychiatry. 2020 Jun 30;26(9):5398–406. doi: 10.1038/s41380-020-0829-y (PMC8589678; doi:10.1038/s41380-020-0829-y)
Supplement: Supplementary file 1 — Supplement [file 41380_2020_829_MOESM1_ESM.docx]

**Supplementary Material**

[^11^C]*(R)*-PK11195 BP_ND_ in caudate, amygdala, hippocampus and temporal cortex

[^11^C]*(R)*-PK11195 BP_ND_ was very low in the caudate, amygdala, hippocampus and temporal cortex, with multiple negative values in all groups and mean values of around zero or below (Supplementary Figure S1). This suggests that specific binding in these regions is lower than that of the reference region (cerebellar grey matter), rendering this methodology unsuitable for these regions. We therefore do not present data from these regions.


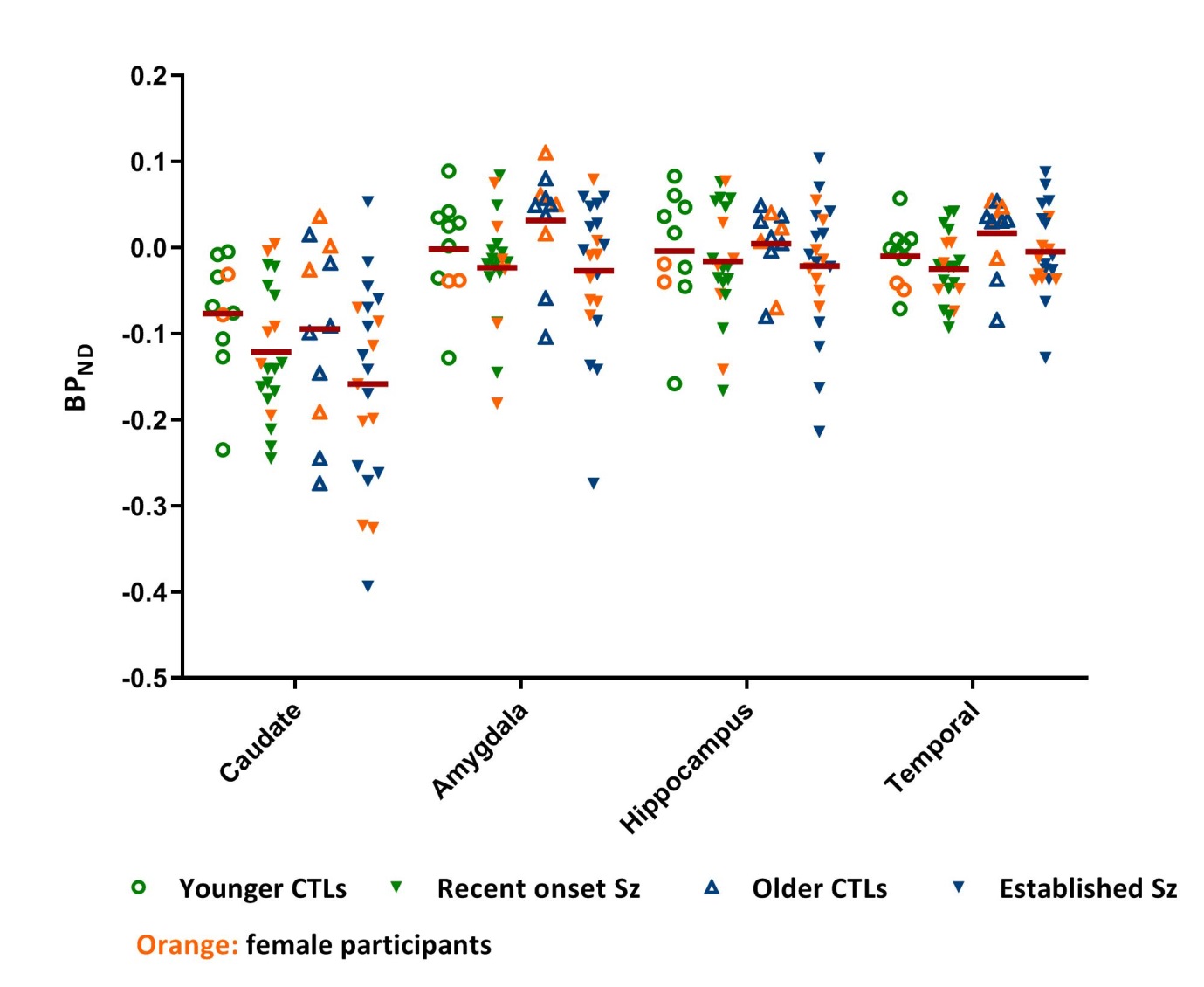


**Supplementary Figure S1** [^11^C]*(R)*-PK11195 binding potential (BP_ND_) in caudate, amygdala, hippocampus and temporal cortex in recent onset and established schizophrenia and age, and sex-matched controls. Orange symbols represent female participants. Horizontal lines in graph indicate mean values.

An overview of psychotropic medication of the patient groups can be found in Supplementary Table S1 and S2.

**Supplementary Table S1** Recent onset Schizophrenia group - Psychotropic medication per patient (ID) as assessed during scan session. OD = once a day, BD = twice a day, mane = in the morning, nocte = at night, PRN = as required, Q4S = every 4 weeks, P.O. = orally.

| **ID** | **Sex** | **Antipsychotic** | **Dose** | **Frequency** | **Route** | **Antidepressant** | **Dose** | **Frequency** | **Route** | **Other** | **Dose** | **Frequency** | **Route** |
| --- | --- | --- | --- | --- | --- | --- | --- | --- | --- | --- | --- | --- | --- |
| 1 | Male | risperidone | 2mg | BD | P.O. |  |  |  |  |  |  |  |  |
| 2 | Male | quetiapine | 50mg | OD | P.O. |  |  |  |  |  |  |  |  |
| 3 | Female | aripiprazole | 10mg | nocte | P.O. |  |  |  |  |  |  |  |  |
| 4 | Female | n/a | n/a | n/a | n/a | citalopram | 40mg | OD | P.O. |  |  |  |  |
| 5 | Male | olanzapine | 5mg | OD | P.O. |  |  |  |  |  |  |  |  |
| 6 | Male | n/a | n/a | n/a | n/a |  |  |  |  |  |  |  |  |
| 7 | Male | olanzapine | 5mg | OD | P.O. |  |  |  |  |  |  |  |  |
| 8 | Male | risperidone | 3mg | OD | P.O. |  |  |  |  |  |  |  |  |
| 9 | Male | n/a | n/a | n/a | n/a | mirtazapine | 30mg | OD | P.O. |  |  |  |  |
| 10 | Male | olanzapine | 10mg | OD | P.O. |  |  |  |  |  |  |  |  |
| 11 | Male | olanzapine | 20mg | OD | P.O. |  |  |  |  |  |  |  |  |
| 12 | Female | risperidone | 3mg | OD | P.O. |  |  |  |  |  |  |  |  |
| 13 | Male | aripiprazole | 5mg | mane | P.O. |  |  |  |  |  |  |  |  |
|  |  | olanzapine | 10mg | OD | P.O. |  |  |  |  |  |  |  |  |
| 14 | Female | n/a | n/a | n/a | n/a | amitriptyline | 80-90mg | nocte | P.O. |  |  |  |  |
| 15 | Male | aripiprazole | 15mg | OD | P.O. |  |  |  |  |  |  |  |  |
| 16 | Female | propranolol | 80mg | nocte | P.O. | sertraline | 150mg | nocte | P.O. | diazepam | 2mg | PRN | P.O. |
|  |  | aripiprazole | 10mg | nocte | P.O. |  |  |  |  |  |  |  |  |
| 17 | Male | n/a | n/a | n/a | n/a | fluoxetine | 20mg | OD | P.O. |  |  |  |  |
| 18 | Male | aripiprazole | 20mg | OD | P.O. |  |  |  |  |  |  |  |  |
| 19 | Female | aripiprazole | 10mg | OD | P.O. |  |  |  |  |  |  |  |  |
| 20 | Male | n/a | n/a | n/a | n/a | mirtazapine | 45mg | nocte | P.O. |  |  |  |  |
|  |  |  |  |  |  | fluoxetine | 20mg | OD | P.O. |  |  |  |  |

**Supplementary Table S2** Established Schizophrenia group- Psychotropic medication per patient (ID) as assessed during scan session. - OD = once a day, BD = twice a day, mane = in the morning, nocte = at night, PRN = as required, Q4S = every 4 weeks, Q2S = every 2 weeks, P.O. = orally, IM = intramuscular (including depots).

| **ID** | **Sex** | **Antipsychotic** | **Dose** | **Frequency** | **Route** | **Antidepressant** | **Dose** | **Frequency** | **Route** | **Other** | **Dose** | **Frequency** | **Route** |
| --- | --- | --- | --- | --- | --- | --- | --- | --- | --- | --- | --- | --- | --- |
| 1 | Male | quetiapine | 200mg | BD | P.O. | mirtazapine | 45mg | nocte | P.O. |  |  |  |  |
| 2 | Male | flupentixol decanoate | 60mg | Q4S | IM |  |  |  |  |  |  |  |  |
| 3 | Male | sulpiride | 200mg | BD | P.O. | fluoxetine | 60mg | OD | P.O. |  |  |  |  |
| 4 | Male | olanzapine | 20mg | OD | P.O. |  |  |  |  |  |  |  |  |
| 5 | Female | flupentixol decanoate | 80mg | Q4S | IM |  |  |  |  |  |  |  |  |
| 6 | Male | olanzapine | 20mg | nocte | P.O. | sertraline | 100mg | OD | P.O. |  |  |  |  |
| 7 | Male | aripiprazole | 30mg | mane | P.O. | venlafaxine | 225mg | mane | P.O. | zopiclone | 7.5mg | nocte | P.O. |
| 8 | Male | amisulpride | 200mg | OD | P.O. | lofepramine | 210mg | OD | P.O. | propranolol MR | 80mg | BD | P.O. |
| 9 | Female | clozapine | 300mg | nocte | P.O. |  |  |  |  |  |  |  |  |
|  |  | amisulpride | 200mg | BD | P.O. |  |  |  |  |  |  |  |  |
| 10 | Female | aripiprazole | 7.5mg | nocte | P.O. |  |  |  |  | zopiclone | 3.75mg | PRN | P.O. |
| 11 | Male | haloperidol decanoate | 50mg | Q4S | IM |  |  |  |  |  |  |  |  |
| 12 | Male | risperdal consta | 50mg | Q2S | IM |  |  |  |  |  |  |  |  |
| 13 | Female | zuclopenthixol decanoate | 200mg | Q2S | IM |  |  |  |  |  |  |  |  |
|  |  |  |  |  |  |  |  |  |  | diazepam | 2mg | PRN | P.O. |
| 14 | Female | clozapine | 100mg 200mg | Mane  Nocte | P.O. | sertraline | 50mg | mane | P.O. |  |  |  |  |
|  |  | aripiprazole | 15mg | mane | P.O. |  |  |  |  |  |  |  |  |
| 15 | Male | chlorpromazine | 50mg | mane | P.O. |  |  |  |  |  |  |  |  |
|  |  | flupentixol decanoate | 100mg | Q2S | IM |  |  |  |  |  |  |  |  |
| 16 | Female | quetiapine | 25-50mg | nocte | P.O. | imipramine | 50mg | nocte | P.O. |  |  |  |  |
| 17 | Male | amisulpride | 500mg | BD | P.O. | sertraline | 50mg | mane | P.O. |  |  |  |  |
| 18 | Male | aripiprazole | 30mg | OD | P.O. | venlafaxine | 75mg | OD | P.O. | pregabalin | 150mg | OD | P.O. |
|  |  | flupentixol | 3mg | OD | P.O. |  |  |  |  | zopiclone | 7.5mg | PRN | P.O. |
| 19 | Male | flupentixol decanoate | 40mg | Q2S | IM |  |  |  |  |  |  |  |  |
| 20 | Female | clozapine | 125mg 225mg | Mane  Nocte | P.O. |  |  |  |  | pregabalin | 75mg | BD | P.O. |
| 21 | Female | olanzapine | 17.5mg | nocte | P.O. | citalopram | 20mg | mane | P.O. |  |  |  |  |
|  |  | aripiprazole | 5mg | mane | P.O. |  |  |  |  |  |  |  |  |

Regional [^11^C]*(R)*-PK11195 BP_ND_ in schizophrenia compared to controls in individual ROIs

Exploratory *post hoc* independent samples *t*-tests (2-tailed) were carried out to test the significance of any difference in BP_ND_ in individual ROIs. Furthermore, the effect size of between-group differences in BP_ND_ was calculated as Cohen’s *d* (the difference between the means divided by the pooled standard deviation [Supplementary Table S3]). The data showed that BP_ND_ was lower in recent onset patients (n=20) compared to controls (n=10) in all ROIs except the brainstem. BP_ND_ was higher in established patients (n=21) than controls (n=10) in ACC, PFC and parietal cortex. BP_ND_ was lower in established patients compared to controls in all other regions.

**Supplementary** **Table S3** Regional [^11^C]*(R)*-PK11195 BP_ND_ in recent onset and established schizophrenia compared to separate age- and sex-matched healthy controls. BP_ND_ values are presented as mean±SD. BP_ND_ = binding potential, ROI = region of interest.

|  | **Recent onset patients** | | | | |  |
| --- | --- | --- | --- | --- | --- | --- |
|  | Controls (n=10) | Patients (n=20) | CTLs vs Patients | | |  |
| **Region** | BP_ND_ | BP_ND_ | Difference | |  | Cohen’s *d* |
| ACC | 0.086±0.038 | 0.059±0.064 | -37% |  | | 0.51 |
| PFC | 0.058±0.042 | 0.036±0.064 | -47% |  | | 0.41 |
| OFC | 0.039±0.035 | 0.011±0.058 | -112% |  | | 0.59 |
| Parietal | 0.044±0.041 | 0.033±0.047 | -29% |  | | 0.25 |
| Putamen | 0.075±0.047 | 0.037±0.060 | -43% |  | | 0.71 |
| Thalamus | 0.196±0.063 | 0.174±0.065 | -12% |  | | 0.35 |
| Brainstem | 0.142±0.061 | 0.146±0.063 | 3% |  | | 0.07 |
| Mean all ROIs | 0.092±0.030 | 0.071±0.050 | -23% |  | | 0.51 |
|  | **Established patients** | | | | |  |
|  | Controls (n=11) | Patients (n=21) | CTLs vs Patients | | |  |
| **Region** | BP_ND_ | BP_ND_ | Difference |  | | Cohen’s *d* |
| ACC | 0.147±0.076 | 0.149±0.085 | 1% |  | | 0.03 |
| PFC | 0.083±0.055 | 0.084±0.082 | 1% |  | | 0.92 |
| OFC | 0.108±0.060 | 0.092±0.070 | -16% |  | | 0.25 |
| Parietal | 0.030±0.045 | 0.076±0.056 | 87% |  | | 0.91 |
| Putamen | 0.118±0.041 | 0.080±0.064 | -38% |  | | 0.71 |
| Thalamus | 0.219±0.060 | 0.160±0.095 | -31% |  | | 0.74 |
| Brainstem | 0.149±0.047 | 0.133±0.064 | -11% |  | | 0.29 |
| Mean all ROIs | 0.122±0.044 | 0.111±0.062 | -9% |  | | 0.21 |

Inspection of the data shows higher BP_ND_ in established patients (n=21) compared to recent onset (n=20) in ACC, PFC, OFC and parietal cortex (Supplementary Figure S2). However, in the ACC, PFC, OFC, AND putamen BP_ND_ appears higher in older controls compared to younger, suggesting this is related to increased age. This is confirmed by two-way ANOVA for these regions (Supplementary Table S4). Supplementary Table S4 also shows that in the parietal cortex there is no main effect of age, but a significant age X diagnosis interaction, suggesting that the higher BP in the parietal cortex in established patients compared to recent onset patients is due to illness stage.


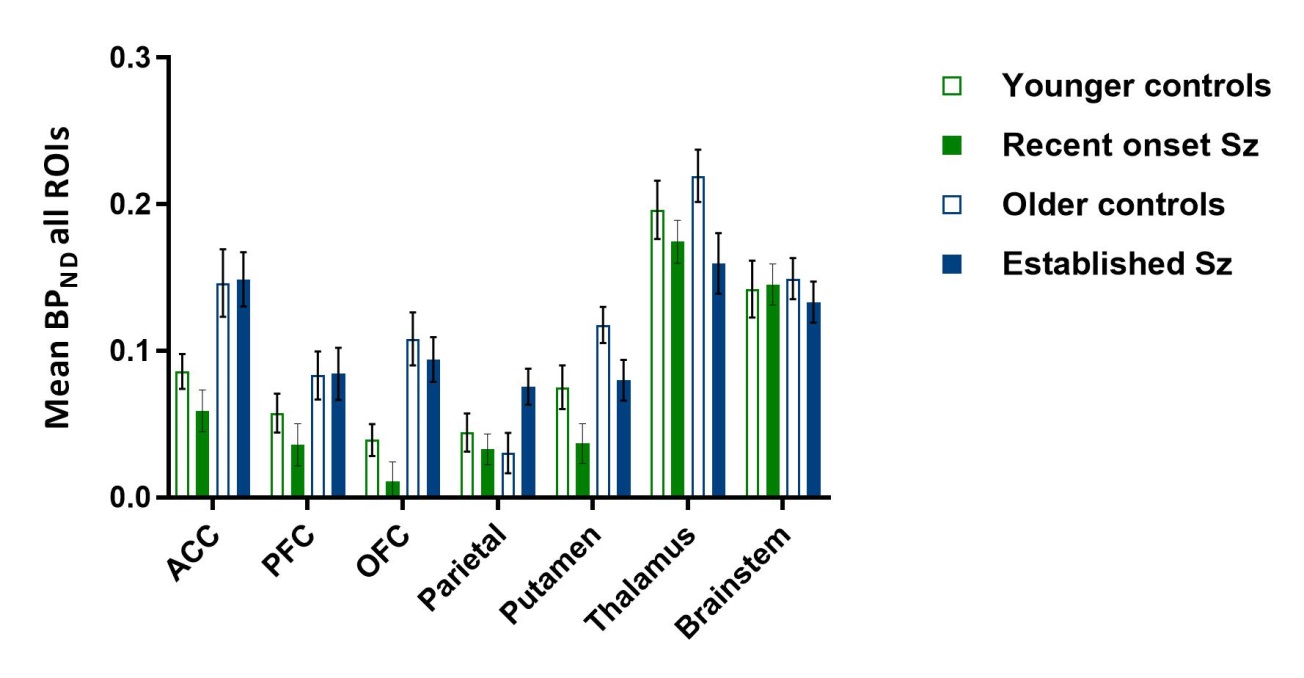


**Supplementary Figure S2** [^11^C]*(R)*-PK11195 BP_ND_ for individual ROIs in recent onset and established schizophrenia compared to separate age- and sex-matched healthy controls.

**Supplementary** **Table S4** Overview of comparison of binding potential in the regions of interest in age group (young versus old), diagnosis (schizophrenia patients versus controls), and age X diagnosis interaction. * = statistically significant at <0.05. Sz = schizophrenia patients.

|  | **Age**  Young vs old | | **Diagnosis**  Sz vs controls | | **Age X Diagnosis** | |
| --- | --- | --- | --- | --- | --- | --- |
|  | *F* | *p* | *F* | *p* | *F* | *p* |
| ACC | 15.49 | 0.0002* | 0.41 | 0.53 | 0.59 | 0.45 |
| PFC | 4.28 | 0.04* | 0.33 | 0.57 | 0.41 | 0.53 |
| OFC | 22.18 | 0.00002* | 1.70 | 0.20 | 0.19 | 0.67 |
| Parietal | 1.18 | 0.28 | 1.63 | 0.21 | 4.61 | 0.04* |
| Putamen | 7.89 | 0.01* | 6.24 | 0.02* | 0.00 | 1.00 |
| Thalamus | 0.05 | 0.83 | 4.03 | 0.049* | 0.88 | 0.35 |
| Brainstem | 0.02 | 0.88 | 0.16 | 0.69 | 0.35 | 0.56 |

Comparing all patients combined with all controls combined (Supplementary Figure S3 and Table S5), mean BP_ND_ in schizophrenia was slightly lower than in controls in all regions except the parietal cortex.


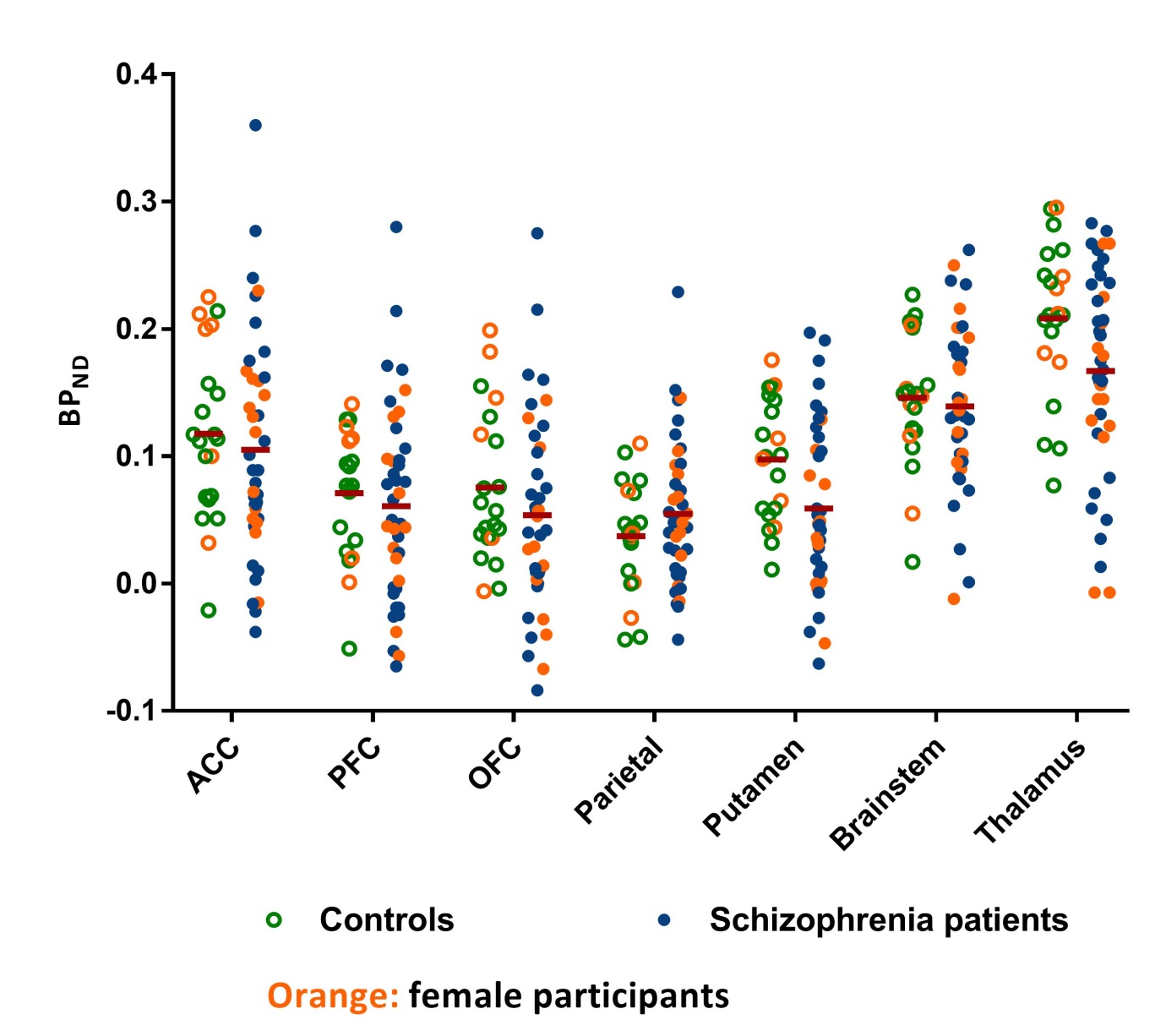


**Supplementary Figure S3** [^11^C]*(R)*-PK11195 binding potential (BP_ND_) values for individual regions of interest in all schizophrenia patients compared to age- and sex-matched controls. Orange symbols represent female participants. Horizontal lines in graph indicate mean values.

**Supplementary Table S5** Regional [^11^C]*(R)*-PK11195 binding potential (BP_ND_) in all schizophrenia patients compared to age- and sex-matched healthy controls. BP_ND_ values are presented as mean±SD. ROI = region of interest, Sz = schizophrenia.

|  | **Controls (n=21)** | **Patients with Sz (n=41)** | **CTLs vs Patients** | |
| --- | --- | --- | --- | --- |
| **Region** | BP_ND_ | BP_ND_ | Difference | Cohen’s *d* |
| ACC | 0.118±0.067 | 0.105±0.087 | -12% | 0.17 |
| PFC | 0.071±0.050 | 0.061±0.076 | -15% | 0.16 |
| OFC | 0.075±0.060 | 0.054±0.076 | -33% | 0.31 |
| Parietal | 0.037±0.043 | 0.055±0.056 | 39% | 0.36 |
| Putamen | 0.098±0.048 | 0.059±0.065 | -50% | 0.68 |
| Thalamus | 0.208±0.608 | 0.167±0.081 | -22% | 0.10 |
| Brainstem | 0.146±0.053 | 0.139±0.063 | -5% | 0.12 |
| Mean all ROIs | 0.108±0.039 | 0.091±0.057 | -16% | 0.35 |

Exploring the effects of antipsychotic medication on regional [^11^C]*(R)*-PK11195 BP_ND_ in recent onset patients

Based on previous work from our group,^1^ *post hoc* tests were carried out to explore any effects of antipsychotic medication on BP_ND_. Patients with recent onset schizophrenia who were antipsychotic-free (n=6) were compared with those who were currently taking an antipsychotic (n=14). Of the 6 antipsychotic-free patients, 2 were antipsychotic-naïve and 4 had been antipsychotic-free for a mean of 94 days prior to scanning (range 6 days- 1.5 years) with a mean lifetime antipsychotic exposure of 68 days (range 56-91 days). Patients currently taking an antipsychotic had a mean lifetime exposure of 88 days (range 51-143) at the time of scanning (Supplementary Table S6).

**Supplementary Table S6** Patient characteristics for antipsychotic-free and medicated recent onset patients, and healthy younger controls. Values are presented as mean±SD. AP = antipsychotic, BMI = Body Mass Index, PANSS = Positive and Negative Syndrome Scale, PSP = Personal and Social Performance Scale.

|  | **Recent** |  |  |  | **CTLs vs**  **AP-free** |
| --- | --- | --- | --- | --- | --- |
| **Characteristics** | AP-free  (n= 6) | Medicated  (n=14) | *p* | Younger Controls  (n=10 ) | *p* |
| Gender (M) | 4 (67%) | 10 (71%) | - |  | - |
| Age (years) | 22 ± 4 | 25 ± 6 | 0.29 | 26 ± 4 | 0.14 |
| BMI (kg/m^2^) | 23.8 ± 2.6 | 25.0 ± 4.0 | 0.51 | 26.5 ± 3.6 | 0.13 |
| Smoking (Yes) | 6 (100%) | 11 (79%) | - | 4 (40%) | - |
| Duration of illness (months) | 12 ± 11 | 17 ± 19 | 0.58 | - | - |
| PANSS total | 52 ± 10 | 58 ± 12 | 0.27 | - | - |
| PANSS positive | 14 ± 6 | 15 ± 6 | 0.69 | - | - |
| PANSS negative | 10 ± 3 | 13 ± 5 | 0.28 | - | - |
| PANSS general | 28 ± 5 | 30 ± 7 | 0.36 | - | - |
| PSP | 66 ± 11 | 57 ± 15 | 0.18 | - | - |
| Injected mass (μg) | 1.4 ± 0.4 | 2.1 ± 2.0 | 0.44 | 1.4 ± 0.6 | 0.98 |

There were no statistically differences in BP_ND_ between antipsychotic-exposed patients and their recent onset controls (Supplementary Table S7).

**Supplementary Table S7** Regional [^11^C]*(R)*-PK11195 binding potential (BP_ND_) in medicated recent onset schizophrenia patients compared to age- and sex-matched healthy controls (CTLs). BP_ND_ values are presented as mean±SD. ROI = region of interest, Sz = schizophrenia.

|  | **Controls (n=10)** | **Medicated Sz (n=14)** | **CTLs vs Medicated** | |
| --- | --- | --- | --- | --- |
| **Region** | BP_ND_ | BP_ND_ | Difference | Cohen’s *d* |
| ACC | 0.086±0.038 | 0.069±0.060 | -22% | 0.34 |
| PFC | 0.058±0.042 | 0.041±0.064 | -34% | 0.31 |
| OFC | 0.039±0.035 | 0.016±0.058 | -84% | 0.48 |
| Parietal | 0.044±0.041 | 0.039±0.046 | -12% | 0.12 |
| Putamen | 0.075±0.047 | 0.045±0.066 | -24% | 0.52 |
| Thalamus | 0.196±0.063 | 0.166±0.054 | -17% | 0.51 |
| Brainstem | 0.142±0.061 | 0.188±0.058 | 28% | 0.77 |
| Mean all ROIs | 0.092±0.030 | 0.081±0.046 | -12% | 0.24 |

Inspection of the data for individual ROIs (Supplementary Figure S4b, Supplementary Table S8) showed that BP_ND_ was higher in antipsychotic-exposed compared to antipsychotic-free patients in all ROIs. Independent samples *t-*tests showed this was statistically significant in the brainstem (*p*=0.018). There was homogeneity of variances in each ROI, as assessed by Levene’s test.


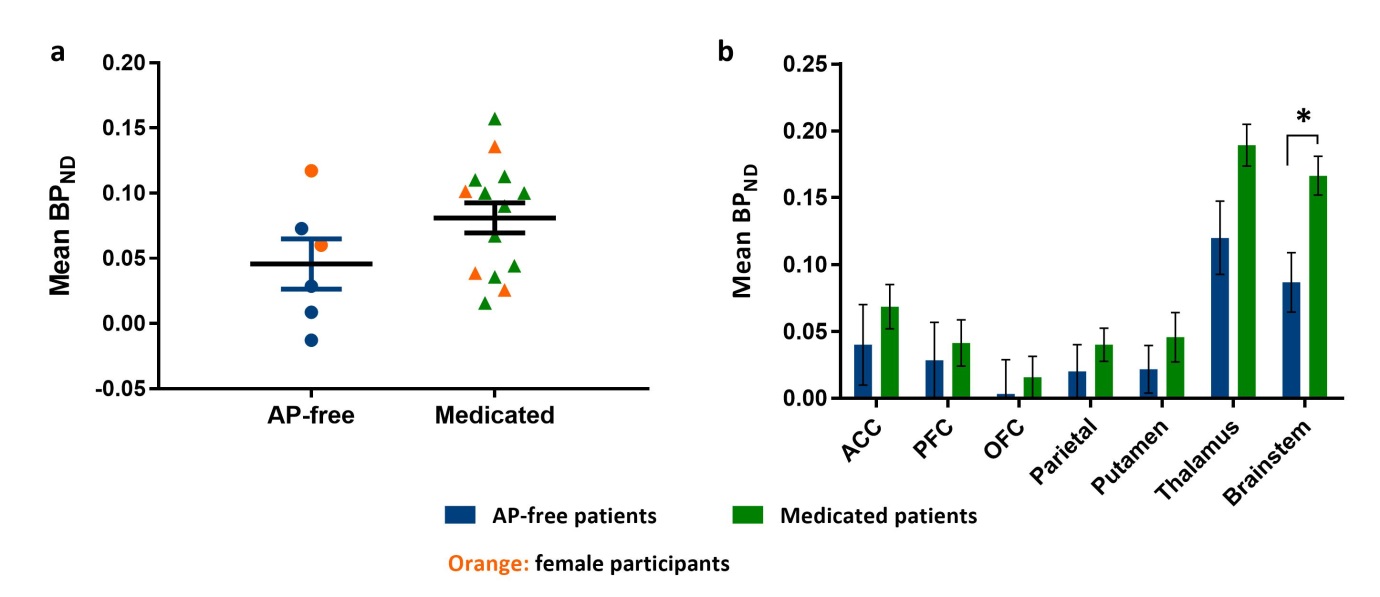


**Supplementary Figure S4** [^11^C]*(R)*-PK11195 binding potential (BP_ND_) in antipsychotic (AP)-free and medicated patients with recent onset schizophrenia. (a) Mean binding potential (BP_ND_) across all regions of interest (ROI). (b) Mean BP_ND_ for individual ROIs. Orange symbols represent female participants. * = significant difference between AP-free and medicated patients at *p=*0.018.

**Supplementary Table S8** Regional [^11^C]*(R)*-PK11195 BP_ND_ in Antipsychotic-free compared to medicated recent onset patients. BP_ND_ values are presented as mean±SD. BP_ND_ = binding potential, ROI = region of interest.

|  | **AP-free (n=6)** | **Medicated (n=14)** | **AP-free vs Medicated** | |
| --- | --- | --- | --- | --- |
| **Region** | BP_ND_ | BP_ND_ | Difference | Cohen’s *d* |
| ACC | 0.036±0.073 | 0.069±0.060 | 63% | 0.49 |
| PFC | 0.024± 0.070 | 0.041±0.064 | 52% | 0.25 |
| OFC | 0.0003±0.064 | 0.016±0.058 | 193% | 0.26 |
| Parietal | 0.020±0.052 | 0.039±0.046 | 64% | 0.39 |
| Putamen | 0.018±0.042 | 0.045 ±0.066 | 86% | 0.49 |
| Thalamus | 0.144±0.077 | 0.188 ±0.058 | 27% | 0.65 |
| Brainstem | 0.097±0.058 | 0.166 ±0.054 | 53% | 1.23 |
| Mean all ROIs | 0.048±0.046 | 0.081±0.046 | 69% | 0.72 |

Correlation of regional BP_ND_ with symptom severity

In the established patients, there was a moderate negative correlation between PANSS positive score and BP_ND_ in all ROIs except in the brainstem, reaching significance in the ACC, PFC, OFC and putamen (Supplementary Table S9). A positive correlation was seen in PANNS negative scores, reaching significance in the PFC and OFC. PANSS general and total scores and the PSP did not correlate with BP_ND_ in any region (Supplementary Table S9).

**Supplementary Table S9** Correlation between [^11^C]*(R)*-PK11195 BP_ND_ in regions of interest and clinical measures in patients with *established schizophrenia* (n=21). Spearman’s *rho* was used if a measure was not normally distributed based on Shapiro-Wilk’s test and visual inspection of Q-Q plots. PANSS = Positive and Negative Syndrome Scale, PSP = Personal and Social Performance Scale. * = significant correlation at *p*≤0.05*.*

| **Region** | **PANSS subscale vs BP_ND_ correlations (*p*-values)** | | | | **PSP** |
| --- | --- | --- | --- | --- | --- |
|  | **positive** | **negative** | **general** | **total** |  |
| ACC | *r=*-0.46  *p*= 0.04* | *r=*0.40  *p*=0.07 | *r=*-0.21  *p*=0.36 | *r=*-0.19  *p*=0.41 | *r=*0.16  *p*=0.95 |
| PFC | *r=*-0.47  *p*= 0.03* | *r=*0.53  *p*= 0.01* | *r=*-0.24  *p*=0.29 | *r=*-0.19  *p*=0.40 | *r=*0.10  *p*=0.68 |
| OFC | *r=*-0.45  *p*= 0.04* | *r=*0.66  *p*=0.001* | *r=*-0.17  *p*=0.46 | *r=*-0.03  *p*=0.88 | *r=*-0.11  *p*=0.65 |
| Parietal | *r=*-0.33  *p*= 0.15 | *r=*0.31  *p*=0.18 | *r=*-0.36  *p*=0.11 | *r=*-0.26  *p*=0.26 | *r=*0.20  *p*=0.39 |
| Putamen | *r=*-0.54  *p* =0.01* | *r=*0.35  *p*=0.13 | *r=*-0.31  *p*=0.18 | *r=*-0.26  *p*=0.26 | *r=*-0.11  *p*=0.96 |
| Thalamus | *r=-*0.33  *p*=0.15 | *r=*0.36  *p*=0.11 | *r=*-0.19  *p*=0.42 | *r=*-0.06  *p*=0.79 | *r=*0.07  *p*=0.77 |
| Brainstem | *r=*0.06  *p*=0.80 | *r=*0.24  *p*=0.31 | *r=*-0.13  *p*=0.56 | *r=*0.05  *p*=0.82 | *r=*-0.22  *p*=0.34 |

In recent onset schizophrenia (Supplementary Table S10), there was a weak-moderate positive correlation between PANSS positive score and BP_ND_ in cortical regions reaching trend or near trend significance. There was no correlation between BP_ND_ and PANSS negative score or the PSP. Furthermore, there was a significant moderate positive correlation between BP_ND_ and PANSS general score in the putamen only (*p*=0.046), as well as between PANSS total score and BP_ND_ in the putamen.

**Supplementary** **Table S10** Correlation between [^11^C]*(R)*-PK11195 BP_ND_ in regions of interest and clinical measures in all patients with *recent onset schizophrenia* (n=20). Spearman’s *rho* was used if a measure was not normally distributed based on Shapiro-Wilk’s test and visual inspection of Q-Q plots. PANSS = Positive and Negative Syndrome Scale, PSP = Personal and Social Performance Scale.

| **Region** | **PANSS positive** | **PANSS negative** | **PANSS general** | **PANSS**  **total** | **PSP** |
| --- | --- | --- | --- | --- | --- |
| ACC | *r*=0.36  *p*=0.12 | *r*=0.27  *p*=0.26 | *r*=0.26  *p*=0.28 | *r*=0.35  *p*=0.13 | *r*=-0.06  *p*=0.79 |
| PFC | *r*=0.38  *p*=0.10 | *r*=0.04  *p*=0.87 | *r*=0.18  *p*=0.45 | *r*=0.27  *p*=0.25 | *r*=0.07  *p*=0.77 |
| OFC | *r*=0.37  *p*=0.11 | *r*=0.24  *p*=0.31 | *r*=0.32  *p*=0.17 | *r*=0.40  *p*=0.08 | *r*=-0.07  *p*=0.76 |
| Parietal | *r*=0.39  *p*=0.09 | *r*=0.01  *p*=0.96 | *r*=0.16  *p*=0.49 | *r*=0.23  *p*=0.32 | *r*=-0.03  *p*=0.92 |
| Putamen | *r*=0.27  *p*=0.24 | *r*=0.27  *p*=0.26 | *r*=0.45 *p*=0.046* | *r*=0.44  *p*=0.05* | *r*=-0.06  *p*=0.79 |
| Thalamus | *r*=0.11  *p*=0.65 | *r*=-0.09  *p*=0.69 | *r*=0.27  *p*=0.24 | *r*=0.17  *p*=0.48 | *r*=0.17  *p*=0.49 |
| Brainstem | *r*=-0.04  *p*=0.86 | *r*=0.02  *p*=0.94 | *r*=0.25  *p*=0.29 | *r*=0.17  *p*=0.48 | *r*=0.07  *p*=0.78 |

Taking all patients together (Supplementary Table S11), there was a significant weak-moderate positive correlation between PANSS negative score and BP_ND_ in the ACC, PFC and OFC with a trend in the putamen. There was no correlation between BP_ND_ and PANSS positive, general or total scores or the PSP.

**Supplementary** **Table S11** Correlation between [^11^C]*(R)*-PK11195 BP_ND_ in regions of interest and clinical measures in *all patients with schizophrenia* (n=41). Spearman’s *rho* was used if a measure was not normally distributed based on Shapiro-Wilk’s test and visual inspection of Q-Q plots. PANSS = Positive and Negative Syndrome Scale, PSP = Personal and Social Performance Scale. * = significant correlation at *p*≤0.05*.*

| **Region** | **PANSS positive** | **PANSS negative** | **PANSS general** | **PANSS**  **total** | **PSP** |
| --- | --- | --- | --- | --- | --- |
| ACC | *r=*-0.07  *p*=0.66 | *r=*0.31 *p*=0.045* | *r*=-0.11  *p*=0.48 | *r*=-0.01  *p*=0.96 | *r*=-0.06  *p*=0.69 |
| PFC | *r=*-0.10  *p*=0.55 | *r=*0.32 *p*=0.04* | *r*=-0.12  *p*=0.47 | *r*=-0.02  *p*=0.89 | *r*=0.003 *p*=0.99 |
| OFC | *r=*-0.01  *p*=0.98 | *r=*0.41 *p*=0.01* | *r=*-0.07  *p*=0.66 | *r=*0.07  *p*=0.69 | *r=*-0.17  *p*=0.29 |
| Parietal | *r=*-0.04  *p*=0.79 | *r=*0.17  *p*=0.29 | *r*=-0.19  *p*=0.24 | *r*=-0.07  *p*=0.66 | *r*=0.003 *p*=0.99 |
| Putamen | *r=*-0.14  *p*=0.40 | *r=*0.29  *p*=0.06 | *r*=-0.01  *p*=0.94 | *r*=0.03 *p*=0.848 | *r*=-0.11  *p*=0.49 |
| Thalamus | *r=*-0.12  *p*=0.45 | *r=*0.17  *p*=0.30 | *r*=0.021  *p*=0.90 | *r*=-0.02  *p*=0.91 | *r*=0.14  *p*=0.39 |
| Brainstem | *r=*-0.00  *p*=1.00 | *r=*0.14  *p*=0.39 | *r*=0.07  *p*=0.66 | *r*=0.11  *p*=0.50 | *r*=-0.06  *p*=0.71 |

Choice of radioligand and analytic methods

We chose this tracer because it has the ability to detect changes in TSPO where these are known to exist histologically [1], and because it is not affected by the common polymorphism that affects binding of second-generation radiotracers [2]. Furthermore, a limitation of all studies using TSPO radiotracers is the lack of a reference region due to the ubiquitous presence of TSPO. For comparability with the results from our previous study [3], we preferred to continue to use bilateral cerebellar grey matter to provide as a pseudo-reference tissue input function. This method has been shown to provide a more conservative estimate of BP_ND_ than supervised cluster analysis, another means of approximating a reference region [4], and has been shown to have good validity when compared to the use of an arterial input function [5] negating the need for invasive arterial cannulation. An analysis of the cerebellar BP_ND_ obtained using a data-driven method to extract a reference tissue input function [6] provided reassurance that the study findings are not confounded by a systematic difference in cerebellar TSPO binding between control subjects and patients. Nevertheless, we acknowledge that the use of cerebellar grey matter as a pseudo-reference region may provide lower test-retest reliability for BP_ND_ estimates than the use of arterial input function, as recently demonstrated in healthy controls [7].

**References**

1. Banati RB. Visualising microglial activation in vivo. Glia. 2002;40(2):206-17.

2. Fujita M, Kobayashi M, Ikawa M, Gunn RN, Rabiner EA, Owen DR, et al. Comparison of four (11)C-labeled PET ligands to quantify translocator protein 18 kDa (TSPO) in human brain: (R)-PK11195, PBR28, DPA-713, and ER176-based on recent publications that measured specific-to-non-displaceable ratios. EJNMMI Res. 2017;7(1):84.

3. Holmes SE, Hinz R, Drake RJ, Gregory CJ, Conen S, Matthews JC, et al. In vivo imaging of brain microglial activity in antipsychotic-free and medicated schizophrenia: a [(11)C](R)-PK11195 positron emission tomography study. Mol Psychiatry. 2016;21(12):1672-9.

4. Su Z, Herholz K, Gerhard A, Roncaroli F, Du Plessis D, Jackson A, et al. [(1)(1)C]-(R)PK11195 tracer kinetics in the brain of glioma patients and a comparison of two referencing approaches. Eur J Nucl Med Mol Imaging. 2013;40(9):1406-19.

5. Kropholler MA, Boellaard R, Schuitemaker A, Folkersma H, van Berckel BN, Lammertsma AA. Evaluation of reference tissue models for the analysis of [11C](R)-PK11195 studies. J Cereb Blood Flow Metab. 2006;26(11):1431-41.

6. Turkheimer FE, Edison P, Pavese N, Roncaroli F, Anderson AN, Hammers A, et al. Reference and target region modeling of [11C]-(R)-PK11195 brain studies. J Nucl Med. 2007;48(1):158-67.

7. Plaven-Sigray P, Matheson GJ, Cselenyi Z, Jucaite A, Farde L, Cervenka S. Test-retest reliability and convergent validity of (R)-[(11)C]PK11195 outcome measures without arterial input function. EJNMMI Res. 2018;8(1):102. Epub 2018/12/01.
